# The effect of cobalt on morphology, structure, and ORR activity of electrospun carbon fibre mats in aqueous alkaline environments

**DOI:** 10.3762/bjnano.12.87

**Published:** 2021-10-19

**Authors:** Markus Gehring, Tobias Kutsch, Osmane Camara, Alexandre Merlen, Hermann Tempel, Hans Kungl, Rüdiger-A Eichel

**Affiliations:** 1 Forschungszentrum Jülich GmbH, Institute of Energy and Climate Research – Fundamental Electrochemistry (IEK-9), 52425 Jülich, Germany; 2 Rheinisch-Westfälische Technische Hochschule Aachen, Institute of Physical Chemistry, 52056 Aachen, Germany; 3 IM2NP, CNRS, Aix-Marseille Université, Université de Toulon, Toulon, France

**Keywords:** carbon fibres, cobalt-decorated fibres, electrospinning, metal–air batteries, oxygen reduction

## Abstract

An innovative approach for the design of air electrodes for metal–air batteries are free-standing scaffolds made of electrospun polyacrylonitrile fibres. In this study, cobalt-decorated fibres are prepared, and the influence of carbonisation temperature on the resulting particle decoration, as well as on fibre structure and morphology is discussed. Scanning electron microscopy, Raman spectroscopy, X-ray diffraction, X-ray photoelectron spectroscopy, elemental analysis, and inductively coupled plasma optical emission spectrometry are used for characterisation. The modified fibre system is compared to a benchmark system without cobalt additives. Cobalt is known to catalyse the formation of graphite in carbonaceous materials at elevated temperatures. As a result of cobalt migration in the material the resulting overall morphology is that of turbostratic carbon. Nitrogen removal and nitrogen-type distribution are enhanced by the cobalt additives. At lower carbonisation temperatures cobalt is distributed over the surface of the fibres, whereas at high carbonisation temperatures it forms particles with diameters up to 300 nm. Free-standing, current-collector-free electrodes assembled from carbonised cobalt-decorated fibre mats display promising performance for the oxygen reduction reaction in aqueous alkaline media. High current densities at an overpotential of 100 mV and low overpotentials at current densities of 333 μA·cm^−2^ were found for all electrodes made from cobalt-decorated fibre mats carbonised at temperatures between 800 and 1000 °C.

## Introduction

As the global production of renewable energy is on the rise, demand for sustainable ways of storing this energy in times of overproduction increases [1]. Systems based on abundant, cheap materials with high energy densities are required. Alkaline aqueous metal–air batteries based on zinc or iron anodes are such promising systems due to their high specific energy densities of 
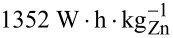
[2] for zinc and 
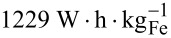
 [2] for iron. However, even with increasing research effort over the past decades, these systems do not live up to their potential; the performance of the cathodes limits the overall performance [3].

The lack of discharge performance is attributed to the sluggish kinetics of the oxygen reduction reaction (ORR) at the air cathode [4], which reduces the practical power density. Further improvements of the cathode are essential for the long-term success of metal–air batteries. Many researchers focus on the active material, that is, the catalyst, which enhances the ORR, and many promising systems have been reported and extensively reviewed [5].

The electrode scaffold receives far less attention. Only few reports elaborate on the importance of the electrode architecture for the catalysts to be utilised to their full potential especially in aqueous metal–air systems. Since initial work by Heise and Schumacher in 1932, pressed layer systems have become a standard [6]. In contrast, quite a number of different approaches are being discussed for non-aqueous metal–air systems, especially the lithium and silicon systems, on account of their different mechanism involving the cathode as an explicit reaction site of the metal redox chemistry [7–9].

From a scientific point of view, in aqueous systems, an air electrode needs to allow gas to diffuse in appropriate amounts towards the active sites, that is, triple-phase contact points. These contact points of air, solid catalyst, and liquid electrolyte, need to be high in number or area. This entails a partial wetting of the electrode to ensure accessibility of the sites for gaseous oxygen. From a more industrial perspective, electrodes intended for mass production need to be composed of cheap materials, ideally from sustainable sources, and to be produced in a way that is scalable.

Carbonised polyacrylonitrile (PAN) fibre mats, which are a promising alternative to pressed carbon powders, are one such approach. These mats are a mechanically stable scaffold for an air electrode with an inherent catalytic activity, due to their nitrogen content [10–12]. The inherent nitrogen leads to nitrogen-doped carbon, which is a known electrocatalyst for the oxygen reduction reaction [13]. The carbonisation temperature determines the degree of graphitisation, the amount of incorporated nitrogen, and the nature of its incorporation [10–12,14]. However, the overall performance of the electrodes is typically not high [15], thus enhancing the activity by addition of dedicated catalytic components is required.

Cobalt and its compounds are known to be electrocatalytically active toward oxygen electrochemistry [16–17]. Multiple ways of introducing cobalt have been reported, and the interaction of cobalt and carbon has been discussed under various aspects since the early 1970s [18]. The applied methods can be generally divided into three main groups: electroplating, electroless plating, and bottom-up methods such as vapour deposition.

Another way to introduce metals to a carbon fibre system in form of nanoparticles was reported by groups who prepared cobalt/cobalt oxide-decorated carbon nanofibres from electrospinning by adding a cobalt salt to the spinning solution [19–22]. Li et al. [19] investigated the activity of the material using a rotating ring disc and dilute 0.1 M KOH electrolyte. They found that the cobalt species were active in both oxygen evolution reaction (OER) and ORR. They also found that increasing amounts of metal additives are detrimental to the mechanical stability of the fibre mat [19], which limits use of fibres with large metal content as a free-standing electrode. In contrast to this, Song et al. [20] investigated the fibres as self-standing electrodes for Li–air batteries using non-aqueous electrolytes in a full-cell setting. They found that cobalt-enhanced fibres displayed an improved cycle life and lower charge (i.e., OER) potential and overall higher capacities during charge and discharge compared to pristine fibres. These properties were attributed to the Co_3_O_4_ nanoparticles present on the fibres. Alegre et al. [21–22] also investigated cobalt-oxide-enhanced fibres, unlike Song et al. they focussed on an alkaline aqueous system. They performed polarisation experiments using their Co_3_O_4_-enhanced fibres as catalytically active component in a pressed carbon electrode in 6 M KOH and found it to be both stable and adequately performing at a current density of 80 mA·cm^−2^. They attributed the enhanced ORR activity (compared to the cobalt-free fibres) to the presence of Co(II) species, graphitic nitrogen, and Co–N_x_ species. They concluded that Co_3_O_4_-enhanced carbon fibres from electrospinning are a convenient and promising material for air electrodes in metal–air batteries. These studies used the cobalt-enhanced fibre material either as a bottom-up catalyst material in aqueous alkaline systems [21–22] or as free-standing electrodes in non-aqueous systems with a lab-scale geometric surface area (smaller than 0.2 cm^2^) [20].

In this study, cobalt-decorated carbon fibre mats are prepared and analysed as self-supporting electrodes of a size suitable for application (3 cm^2^) in half-cells using 6 M KOH electrolyte. The focus of this study lies on the influence of the carbonisation temperature to gain a more detailed understanding of the interactions of the cobalt additive and the carbon framework, as well as the formation and location of cobalt–nitrogen species. The changes in graphitisation, fibre chemistry, and electrochemical ORR performance are studied in depth using scanning electron microscopy (SEM), energy-dispersive X-ray spectroscopy (EDX), X-ray diffraction (XRD), X-ray photoelectron spectroscopy (XPS), Raman spectroscopy, elemental analysis, and inductively coupled plasma optical emission spectrometry (ICP-OES). In addition, the fibres were analysed in terms of their electrochemical activity using linear sweep voltammetry with a focus on the oxygen reduction reaction.

## Experimental

### Synthesis of carbon fibres and electrode preparation

Polyacrylonitrile (PAN) (98%, *M*_W_ = 150,000 g·mol^−1^; BOC sciences, USA) was dissolved in *N*,*N*-dimethyl formamide (DMF) to obtain 10 wt % solutions. Cobalt(II) acetylacetonate (97%, Sigma-Aldrich, Germany) was added to the solution to obtain 1 wt % solutions considering the weight of the cobalt ions in relation to the mass of PAN. The solution was electrospun with the parameters summarised in Table 1. The acceleration voltage was set to 17 kV for cobalt-containing samples and 21 kV for the samples without cobalt additive. The samples were stabilised in air and subsequently carbonised in argon atmosphere for 3 h at temperatures between 600 and 1100 °C. Electrodes were prepared from the carbonised mats by applying a PTFE membrane by means of hot-rolling at 120 °C directly onto the mat without an additional current collector.

**Table 1:** Parameters for the electrospinning process.

Parameter		Value	Unit

temperature		25	°C
rel. humidity		40	%
flow rate		40	μL·min^−1^
nozzle–collector distance		14	cm
collector diameter		6	cm
collector rotation		1500	rpm
voltage (nozzle)	pure PAN	21	kV
	w/ Co additive	17	kV
voltage (collector)		−4	kV

### Physical characterisation

SEM images were recorded using a Quanta FEG 650 (FEI Europe) with an acceleration voltage of 5 kV. The samples were attached to the sample holder using double-sided graphite tape. Conductivity was further improved by applying a copper tape connecting the sample and the graphite tape.

To identify the particles decorating the nanofibres, EDX was performed using an Octane Super EDX detector (EDAX). The programme “monte CArlo SImulation of electroN trajectory in sOlids” (CASINO) [23], which simulates the interaction between electrons and solids was used to choose a suitable acceleration voltage. To differentiate the carbon fibres and the particles an acceleration voltage of 5 kV was employed, allowing for surface analysis. To avoid carbon signal intensity interference from the graphite tape the samples were attached to the sample holder using Cu tape.

X-ray diffractograms were recorded with a PANalytical Empyrean Series 2 (Malvern PANalytical B.V., Netherlands) in Bragg–Brentano geometry at room temperature using a copper X-ray source (λ(Kα_1_) = 1.54056 Å, λ(Kα_2_) = 1.54493 Å). For the measurement, samples of 1 cm^2^ were cut from the fibre mats and fixed on a silicon crystal in a rotating sample holder. The scan range was limited to 10° to 80°, scanned with a step width of 0.01°.

X-ray photoelectron spectroscopy (XPS) was performed with a Phi5000 VersaProbe II (ULVAC-Phi Inc., USA). Spectra were recorded with a resolution of 0.1 eV using Al Kα radiation (1.486 keV) at 50 W and a spot size of 200 μm. Fitting the cobalt XPS spectra follows the suggestions from Biesinger et al. [24] based on the complex multiplet structure of cobalt [25]. Based on these reports the Co 2p range was examined in detail only in the binding energy range from 770 to 795 eV.

Raman spectra were obtained using a Bruker Senterra at room temperature in ambient atmosphere. The excitation wavelength was 532 nm with a power of 2 mW. The signals of two subsequent measurements of 30 s each were added up, to improve the signal-to-noise ratio (co-addition mode). The resulting spectra were analysed by fitting a Lorentz distribution and a Breit–Wigner–Fano distribution, as explained in detail in [12].

For the elemental analysis 2 mg of sample was burned in the elemental analyser (VarioelCube, Elementar) for both CHN and O analyses. The signal was detected using a heat conductivity detector.

ICP-OES for cobalt mass analysis was performed by blending 30 to 50 mg of a sample with 250 mg of a mixture of lithium borates. The blend was heated to 1000 °C within 3 h and kept at this temperature for 30 min. The resulting melt was dissolved in 30 mL of 5% HCl and filled up to a total volume of 50 mL. For each sample two aliquotes of the solution were diluted 1:100 and analysed.

### Electrochemical characterisation

Electrochemical investigation of the electrodes was performed in a FlexCell-PP (Gaskatel, Germany) with a geometric area of the electrode of 3 cm^2^ using 6 M KOH electrolyte (KOH: 85%, VWR, France; deionised water: 0.055 μS·cm^−1^, Purelab flex, Elga Veolia, United Kingdom). The reference electrode was a mercury/mercury oxide electrode (MMO; ALS, Japan) and the counter electrode was a platinum wire. Cells were assembled and equilibrated for 1 h before each measurement.

Subsequent linear sweep voltammetry measurements were performed with a scan rate of 1 mV·s^−1^ in a potential window of 1 V, from 0.5 V vs MMO to −0.5 V vs MMO. The stability tests were performed by applying discharge currents of 6, 12, 18, and 6 mA (2, 4, 6, and 2 mA·cm^−2^) for 36 h.

## Results and Discussion

### Physical characterisation

#### Microscopic analysis of the fibre mats

**Figure 1:**
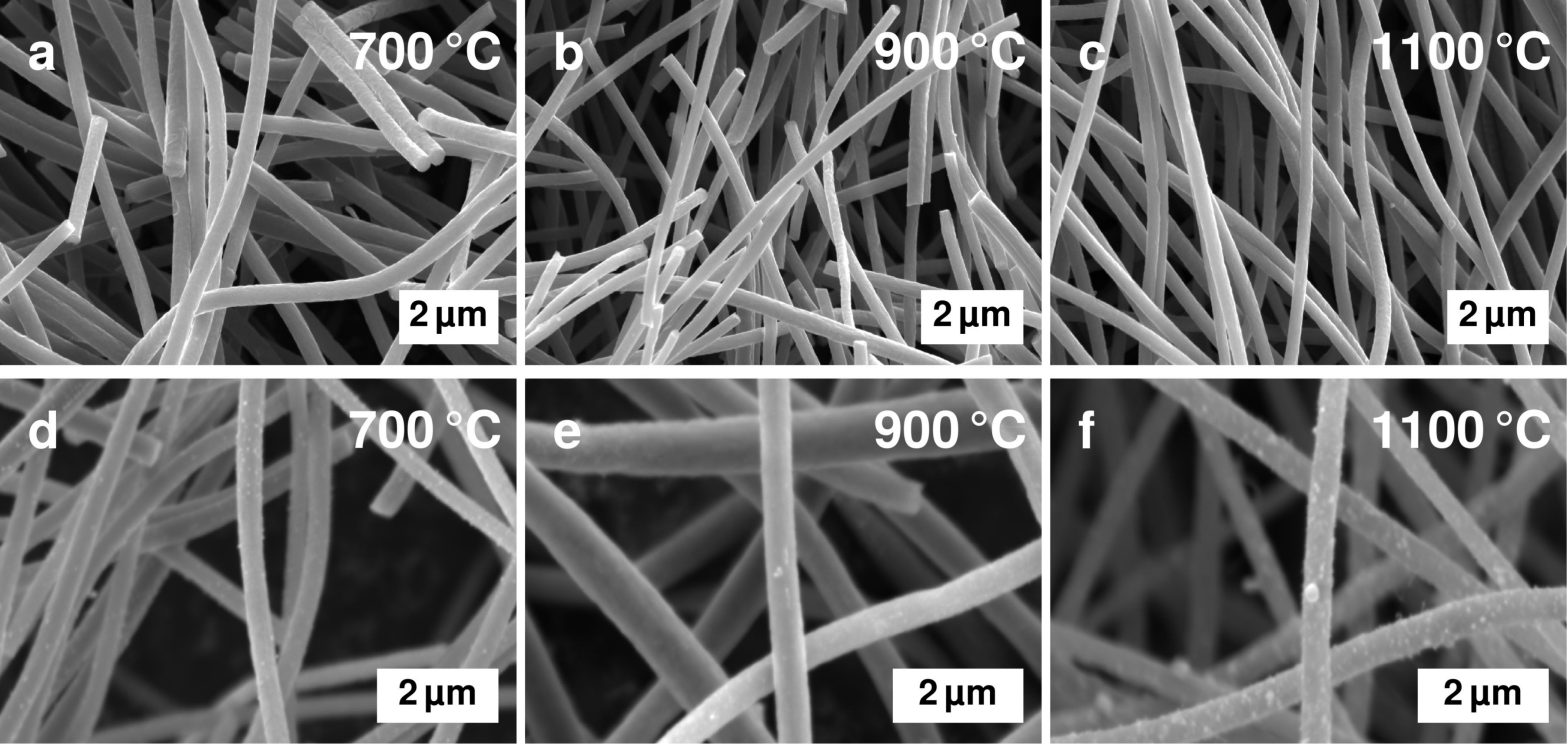
SEM images of (a–c) fibres without cobalt additives and (d–f) fibres with 1.0 wt % Co.

Although previous studies of fibres without additives displayed a decrease in fibre diameter with carbonisation temperature [12], no such trend is visible for the fibres with cobalt from Figure 1. Their diameters range from approximately 200 to 450 nm.

In the sample carbonised at 700 °C (Figure 1d), many very small particles are homogeneously distributed on the surface of the fibres. The fibres of mats carbonised at 1100 °C (Figure 1f) display a relatively homogenous distribution of particles on the surface of the fibres that are larger than the ones visible at lower temperatures.

#### Particle identification

**Figure 2:**
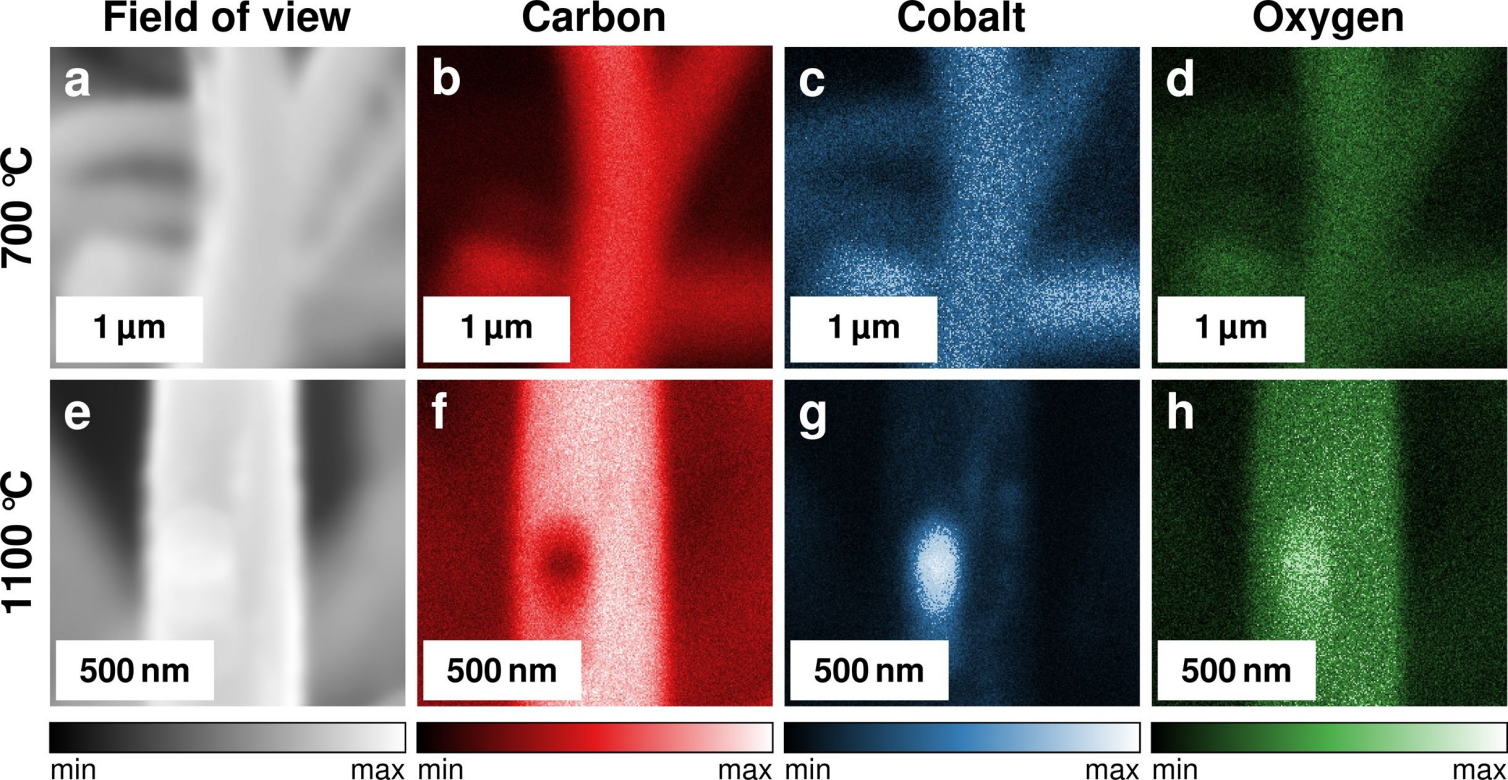
EDX mapping results of a fibre mat containing cobalt, after carbonisation at (a–d) 700 °C and (e–h) 1100 °C. (a, e) SEM reference images, (b, f) carbon K-edge maps, (c, g) cobalt L-edge maps, and (d, h) oxygen K-edge maps.

EDX, as a method of elemental analysis of surfaces on a microscopic scale, was performed to elucidate the cobalt distribution further. EDX maps of fibres carbonised with cobalt at 700 and 1100 °C are shown in Figure 2.

**Figure 3:**
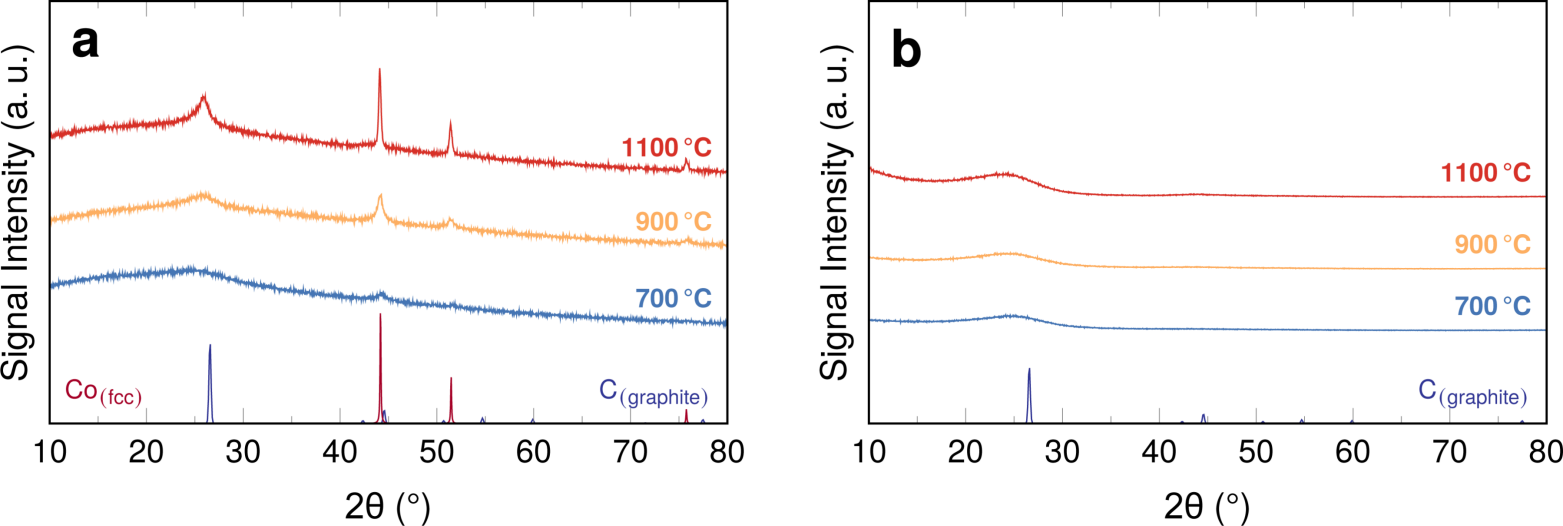
XRD diffractograms of fibres with (a) 1.0 wt % Co and (b) without additives, carbonised at the indicated temperatures. Graphite reference pattern from [26]; reference pattern of fcc Co from [27].

The EDX mapping confirms that, considering the fibre surfaces, the constituting material of the fibres is indeed carbon (Figure 2b and f). The map of the cobalt K-edge for the sample carbonised at 700 °C (Figure 2c) indicates that cobalt is apparently distributed evenly across the entire fibre surfaces. In the sample carbonised at 1100 °C cobalt seems to have agglomerated forming larger particles (Figure 2g). These particles, which are visible in the SEM images as bright spots (cf. Figure 1d–f), are mainly composed of cobalt or contain at least significant amounts of cobalt. To further investigate the nature of the cobalt-containing particles XRD diffractograms were recorded (Figure 3).

**Figure 4:**
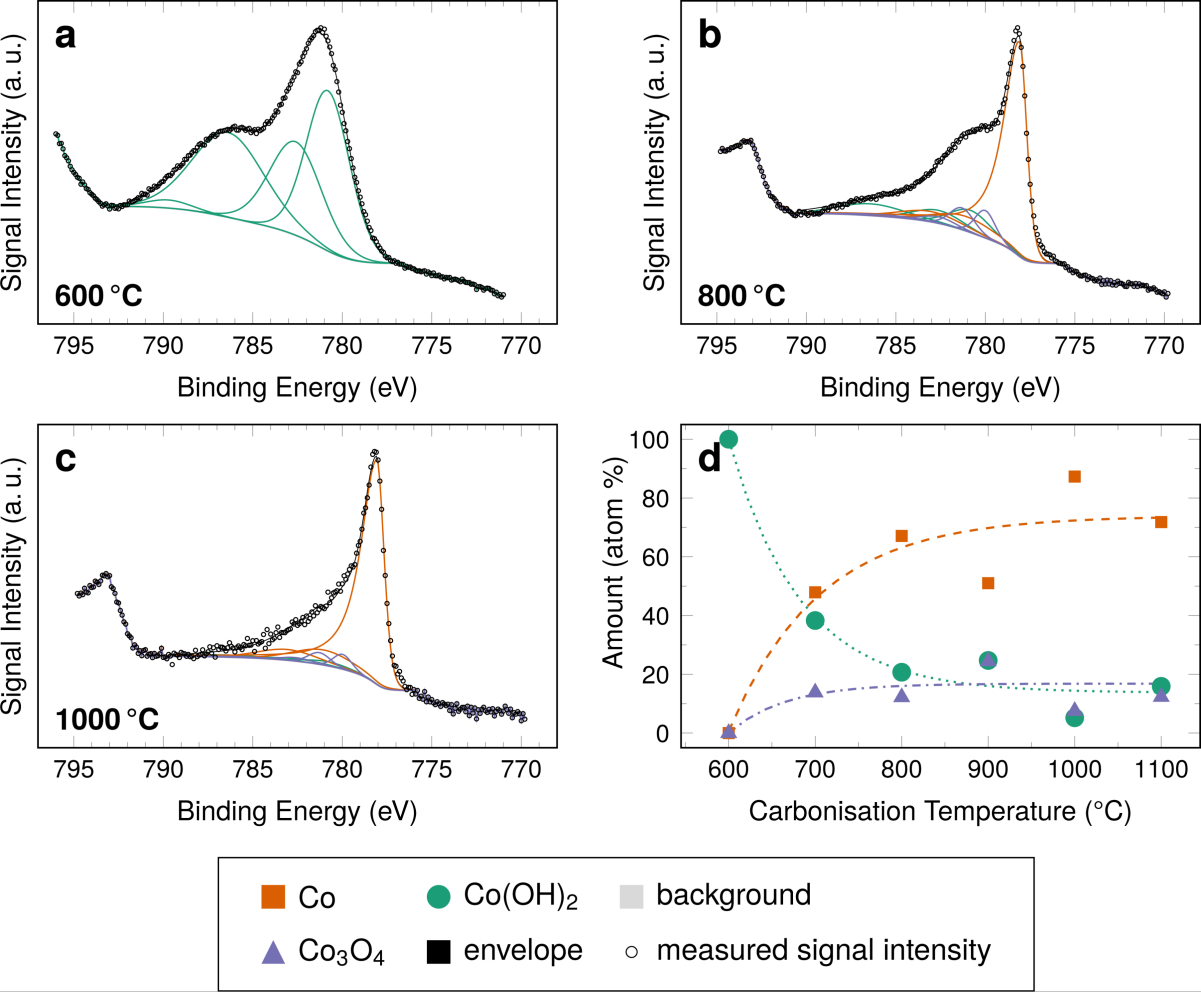
(a–c) Selected Co 2p XPS spectra of samples containing cobalt carbonised at the indicated temperatures. An overview of all spectra is given in Supporting Information File 1. (d) Amount of cobalt species as determined from the area intensity for each species.

The diffractograms for samples with cobalt display the characteristic reflexes at angles attributed to the Co fcc phase. Their intensity increases with carbonisation temperature, which is likely a result of the increasing particle size found in the SEM images. The presence of metallic cobalt as the bulk phase for the particles is clear from this result, but considering the catalytic properties a closer look at the particle surfaces is helpful. Therefore, the Co 2p XPS range was investigated (Figure 4).

The XPS signal of cobalt and its derivatives is non-trivial. Each species, including metallic cobalt, is characterised by peak multiplets. All of these peaks exist in pairs of a 2p_3/2_ peak and a corresponding 2p_1/2_ peak, as was discussed in detail by Biesinger et al. [24] and Gupta and co-workers [25]. The 2p_3/2_ peak multiplets are found in the binding energy range between 775 and 793 eV for the investigated species. Fitting the spectra with peaks attributed to metallic cobalt, Co_3_O_4_, and cobalt(II) species reveals that significant amounts on the particle surfaces are in fact not metallic cobalt but rather cobalt oxides or hydroxides.

The fraction of these species strongly depends on the carbonisation temperature; in the samples carbonised at 600 °C, only a species fitting to the Co(OH)_2_ reference spectra is found on the surface of the fibres. After carbonising at higher temperatures, the amount of the presumed hydroxide decreases, mainly in favour of metallic cobalt and, in parts, cobalt oxide(s). As a general trend, this effect is stronger, when the carbonisation temperatures are higher. The signal intensity of the shoulder of the main Co(0) peak, visible for the sample carbonised at 800 °C, is decreased when the sample is carbonised at 1000 °C.

Cobalt is known to instantaneously oxidise upon contact with air even at room temperature forming an oxide layer with a thickness of 0.8 to 1 nm [28]. Considering the low thickness of the layer and the fact that spontaneous oxidation likely results in amorphous species explains the absence of reflexes attributed to oxidised species, such as Co_3_O_4_, CoO, or Co(OH)_2_, in the XRD diffractograms. This behaviour also explains the plateau in the cobalt metal values found for samples carbonised above 800 °C. At these temperatures the particle surface is completely reduced and only re-oxidised upon contact with air. From an application point of view, this oxide/hydroxide layer may even prove beneficial, as both cobalt [16] and its oxides [29] have been shown to enhance the ORR in alkaline media.

#### Carbon matrix structure

The XRD diffractograms also give insights into the structure of the carbon matrix into which the particles are embedded. The reflex intensity at around 25.9° is characteristic for carbon and, more specifically, graphitic materials based on PAN [30]. Consequently, the diffractograms of the pure PAN fibres (Figure 3b) display a very broad, not very intense reflex around 26°. The reflex is slightly more pronounced in the sample carbonised at 1100 °C compared to the samples carbonised at lower temperatures. This increase of signal intensity with increasing carbonisation temperature indicates an increase in crystalline order, that is, graphitisation, which is in agreement with results for the carbonisation of PAN bulk samples [31].

In the diffractograms of the samples containing cobalt (Figure 3a), the carbon reflex is asymmetric and comparably sharp. The asymmetric and intense reflex may be attributed to the (002) plane spacing of graphitic carbon, which results in a characteristic reflex of relatively high intensity at approximately 26° [31–33]. The formation of a sharp reflex in the presence of cobalt indicates that the cobalt species influences the graphitisation of the material significantly. Indeed, cobalt, iron, and nickel, are known to catalyse the graphitisation process [34–37] with cobalt having the weakest effect [37]. Depending on the cobalt salt the graphitisation process will result in turbostratic carbon [31,35–36]. Turbostratic carbon is typically described as an intermediate between amorphous and graphitic carbon. It is more ordered than amorphous carbon, especially in the short range [33]. However, it lacks long-range order, more specifically a 3D stacking order [35]. The graphitisation effects induced by cobalt and elevated temperatures become more distinct with increasing carbonisation temperatures, that is, the reflex becomes more intense and narrow. It also shifts towards slightly higher angles, which is related to the increasing carbon fraction [38].

**Figure 5:**
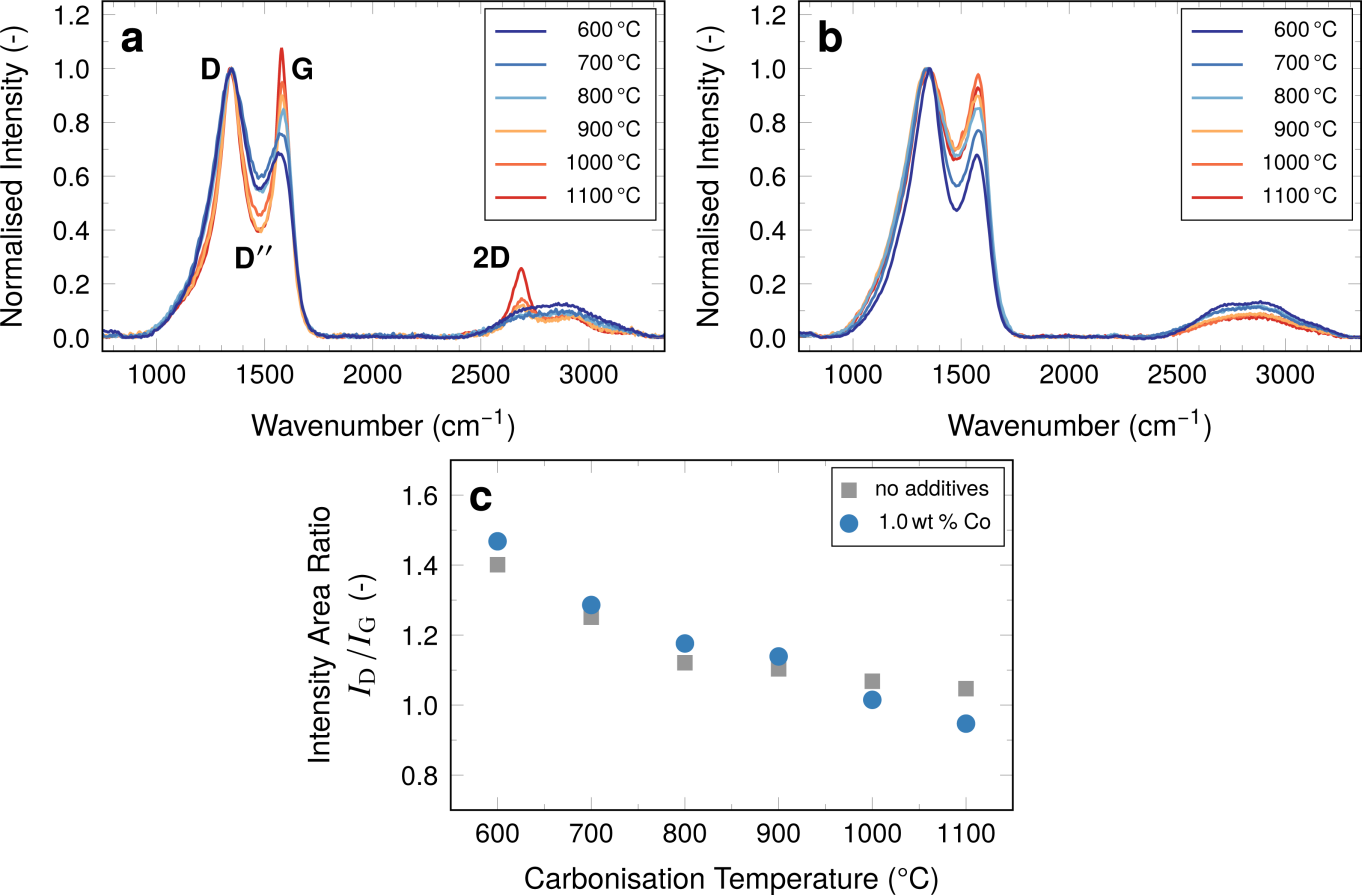
Raman spectra normalised to the height of the D peak for samples with (a) 1.0 wt % Co and (b) without cobalt additives. (c) Development of the *I*_D_/*I*_G_ area ratios.

The formation of graphitic carbon is also reflected in the spectra obtained from Raman spectroscopy of the fibres with and without cobalt. The resulting spectra are shown in Figure 5a,b and the intensity ratio *I*_D_/*I*_G_ of the D peak at 1355 cm^−1^ and the G peak at 1600 cm^−1^, which is an indicator for the degree of graphitisation, is shown in Figure 5c.

For both samples with and without cobalt additives the same general trend for the *I*_D_/*I*_G_ ratio is visible in the graphs. The intensity ratio of the two peaks decreases with increasing carbonisation temperature, as the intensity of the G peak increases relative to the D peak. Overall, the signal intensity ratios decrease from values around 1.4 at 600 °C to around 1.0 at 1100 °C for both samples without additives and samples containing cobalt.

The development of a marked 2D peak at approximately 2680 cm^−1^ has been reported to result from the presence of turbostratic carbon as a type of disorder in graphite [39–40]. Fibres with cobalt display this peak increasingly with higher carbonisation temperatures (Figure 5a), suggesting the formation of turbostratic carbon, especially in samples carbonised at and above 900 °C. This effect is not found in the unmodified fibres (Figure 5b), as suggested by the XRD diffractograms (Figure 3).

Comparing the development of the *I*_D_/*I*_G_ ratios it becomes clear that despite the catalytic effect of cobalt, it plays a significant role only at temperatures above 900 °C. There are two possible explanations for this behaviour. These are the fact that the catalytic effect of cobalt is only moderate [37] or the fact that the effect is limited to the immediate proximity of graphite formation [34]. During carbonisation the cobalt atoms migrate through the material resulting in larger particles, leaving behind the graphite-like turbostratic carbon, the amounts of which are sufficiently within the resolution of the Raman measurement in the samples carbonised above 900 °C (Figure 5a) and above 1000 °C in terms of the *I*_D_/*I*_G_ ratios (Figure 5c).

**Figure 6:**
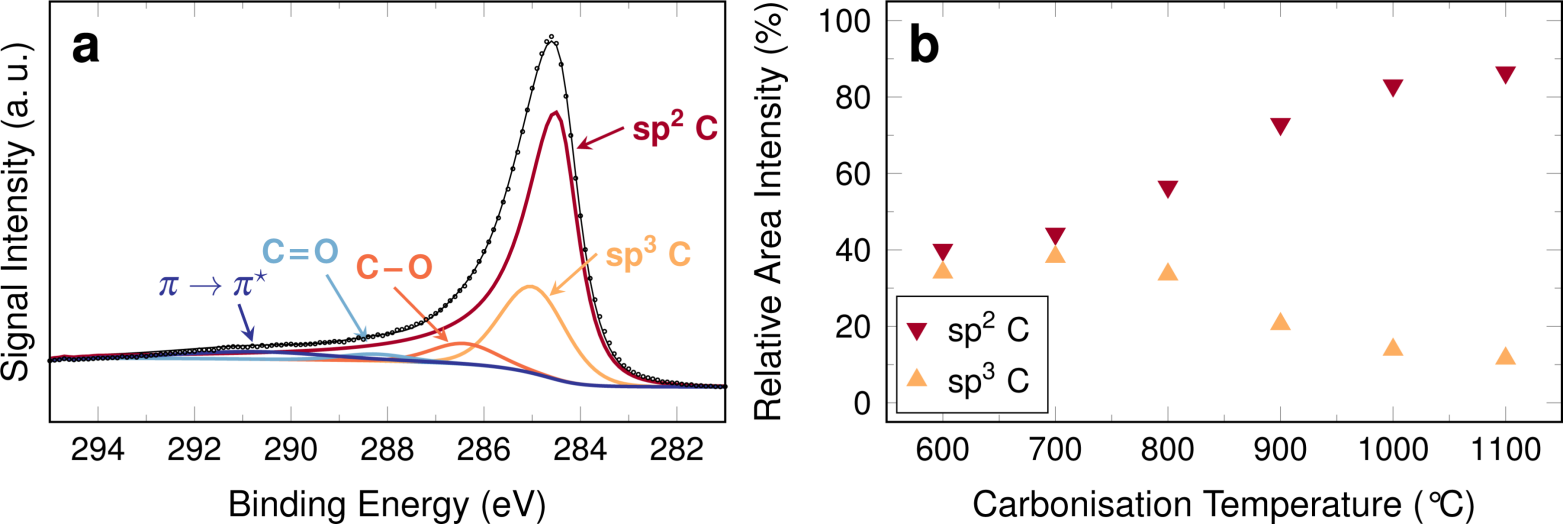
(a) Exemplary XPS C 1s range of a sample containing cobalt carbonised at 900 °C, (b) relative area intensity of sp^2^ and sp^3^ carbon from C 1s XPS spectra of cobalt-decorated fibres. The spectra used for value determination are shown in Supporting Information File 1.

Graphite is based on a two-dimensional carbon structure, which is characterised by the presence of sp^2^ carbon. Indeed, an increasing amount of sp^2^ carbon compared to sp^3^ carbon is found, when the respective signal intensities of the XPS C 1s range of the fibres is considered as shown in Figure 6.

As expected for an increasing graphitisation, the amount of graphitic carbon (sp^2^) increases with carbonisation temperature, while the amount of sp^3^ carbon, expected in more amorphous or polymer-like samples, decreases. In fibres with cobalt, the amount sp^2^ carbon amounts to 87%, which also means that a significant amount of sp^3^ carbon is retained in these samples.

This behaviour is generally in agreement with literature. In coal samples investigated by Manoj and Kunjomana, the formation of turbostratic carbon was found to correlate with content and aromaticity of carbon [32]. An increased aromaticity is implied by an increasing amount of sp^2^ carbon compared to sp^3^ carbon. This would imply that not only the ordering of sp^2^ carbon is increased, but the samples should contain more of it.

Systems without metal additives and those with metal additives can be hardly compared, because of the marked difference in the carbon XPS signal composition. However, even samples without metals have been reported to be converted to graphitic carbon when carbonised at temperatures above 1100 °C [12].

#### Nitrogen-type distribution in presence of cobalt

One of the main reasons why polyacrylonitrile is employed as the precursor material is the fact that it contains nitrogen in significant amounts. The nitrogen is retained in the structure to a certain degree depending on the carbonisation temperature [11–12]. In the previous sections, the influence of the cobalt additives on the graphitisation process and the binding type of carbon have been discussed. It is rather safe to assume that parts of these changes in the carbon binding structure are related to the overall nitrogen content in the system and the way nitrogen is chemically incorporated into the graphite matrix. This incorporation of nitrogen is related closely to the way the cobalt atoms and ions are incorporated into the carbon matrix, which would be relevant for the fraction of cobalt that does not form particles, but remains distributed in the fibres.

**Figure 7:**
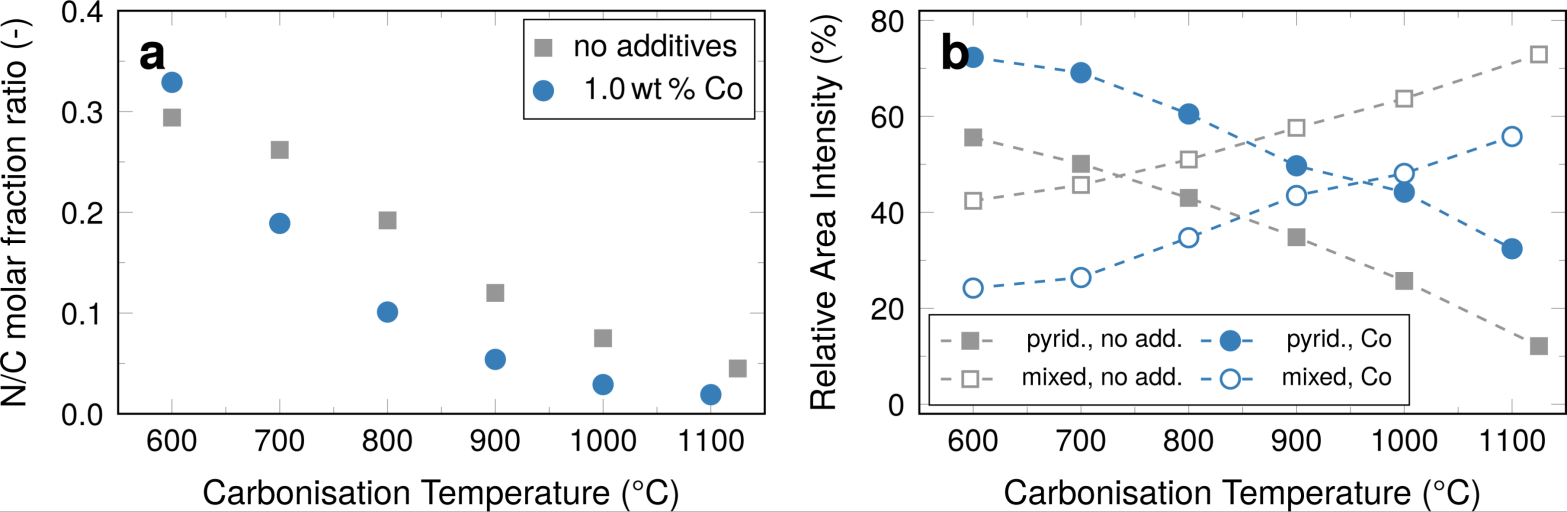
(a) Calculated ratio of molar fractions of nitrogen and carbon and (b) pyridinic nitrogen and the pyrrolic/graphitic nitrogen mixed signal in samples with and without cobalt additives. The corresponding spectra are shown in Supporting Information File 1. Data for unmodified fibres from [12].

The overall composition of the fibres was determined using a combination of elemental analysis (nitrogen and carbon content) and ICP-OES (Figure 7a) and the distribution of nitrogen types was determined by XPS (Figure 7b).

After carbonisation at 1100 °C less than 2 wt % of nitrogen are retained in the fibres in the presence of cobalt. Comparing the fibres with cobalt additive to a reference system without cobalt, it also apparent that cobalt enhances nitrogen removal.

Regardless of the presence of metal species, heteroatom dopants in carbon induce catalytic activity towards the oxygen reduction reaction. However, this activity depends highly on the type of nitrogen bonding. Hence, it is important to examine the way nitrogen is incorporated into the graphitic structure. To this end the XPS spectra were analysed in the relevant nitrogen range. Typically, four nitrogen species should be considered: pyridinic nitrogen at 398.7 eV, pyrrolic nitrogen at 400.3 eV, graphitic nitrogen at 401.2 eV, and pyridinic N-oxides at 402.8 eV [41]. The most important types discussed in the context of the catalytic activity towards the oxygen reduction reaction are pyridinic and graphitic nitrogen [41–44].

The spectra obtained in the measurement did not allow, however, for deconvolution of the pyrrolic N signal and the graphitic N signal for the fibres containing cobalt. Their spectra (see Supporting Information File 1) were thus fitted with only three peaks, considering the middle peak a mixed peak of pyrrolic and graphitic nitrogen. The peak at approximately 398.7 eV was attributed to pyridinic nitrogen, the peak at approximately 402.8 eV to pyridinic N-oxides. To obtain comparable results for the metal-free reference system, the signal intensities of pyrrolic and graphitic nitrogen were summed up for the samples without cobalt.

The general trend of the pyridinic peak area intensity and that of the mixed peak intensity with increasing carbonisation temperature are similar, with the pyridinic peak decreasing and the mixed peak increasing. For fibres with cobalt the relative amount of pyridinic nitrogen decreases from 72% for fibres carbonised at 600 °C to 32% for fibres carbonised at 1100 °C. Likewise, the combined amount of pyrrolic and graphitic nitrogen increases from 24% to 56%. When comparing the unmodified fibres to the fibres with cobalt, the relative signal intensity of pyridinic nitrogen is relatively higher for the latter by about 20%, whereas the combined signal intensity of pyrrolic and graphitic nitrogen is about 20% lower.

It was previously shown that the amount of graphitic nitrogen increases significantly with increasing carbonisation temperatures, while the amount of pyrrolic nitrogen is diminished in fibres without metal additives [12]. It is implied that, qualitatively, the amount of pyrrolic nitrogen decreases as the amount of graphitic nitrogen increases in the samples with cobalt, as the general trend is the same as in the reference system. The point where the system contains less pyridinic nitrogen than combined amounts of pyrrolic and graphitic nitrogen, that is, presumably more active nitrogen positions than inactive ones, is shifted from 700 °C in fibres without cobalt additives to 1000 °C with cobalt. Considering the fact that more nitrogen is removed from the system, as was demonstrated by elemental analysis (Figure 7a) the significance of nitrogen doping in the cobalt-decorated carbon fibres is, however, substantially reduced, in favour of cobalt-based activity.

### Electrochemical ORR characterisation

#### Linear sweep voltammetry using fibre-based electrodes

**Figure 8:**
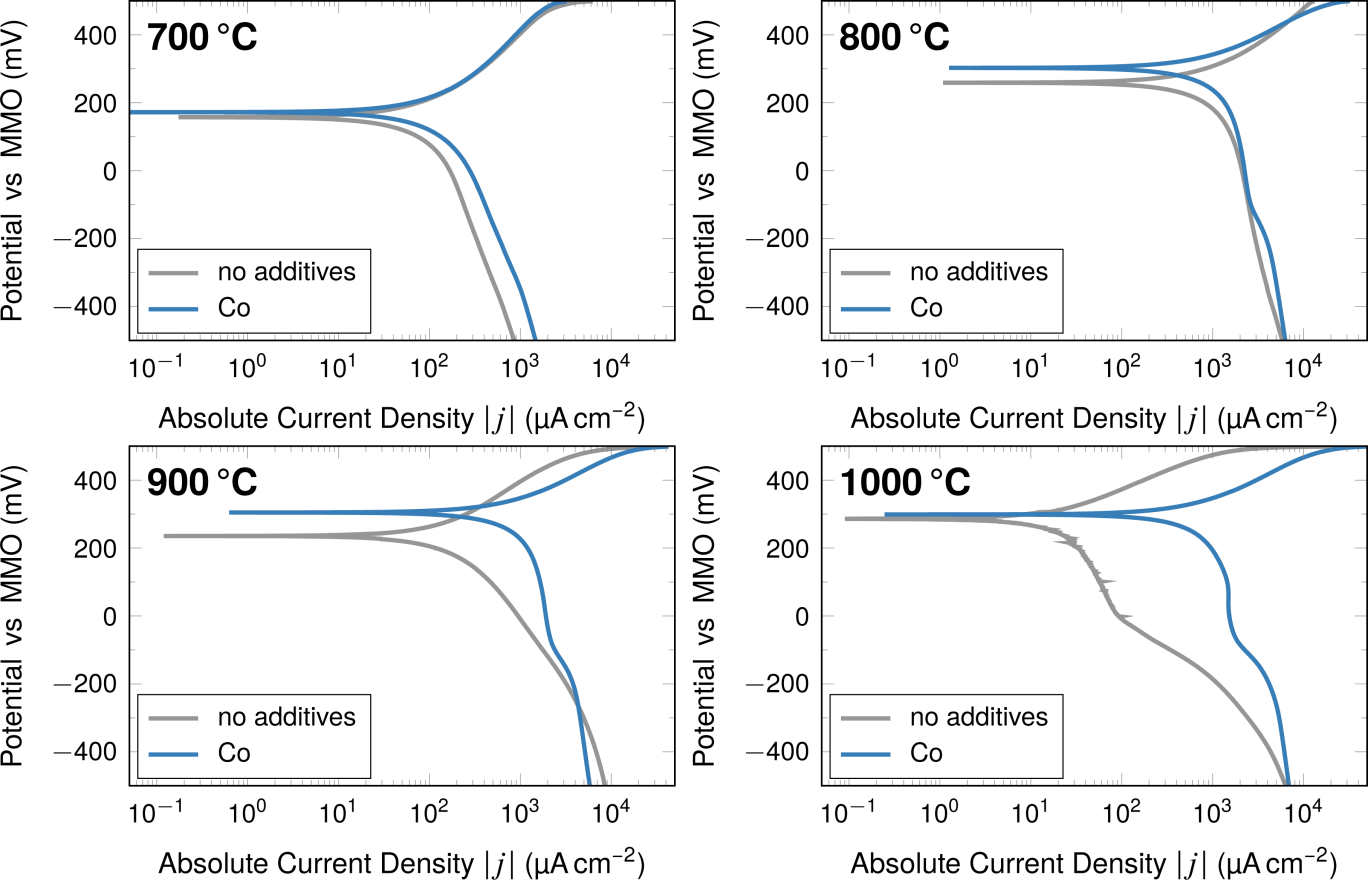
Linear sweep voltammetry measurements of fibre-based electrodes using fibres carbonised at the indicated temperatures.

The oxygen reduction performance of the cobalt-containing system is evaluated using linear sweep voltammetry (LSV). Qualitatively, an improvement is visible for fibres containing cobalt compared to a cobalt-free reference as shown for selected temperatures in Figure 8.

Some general differences as a result of the cobalt enhancement are immediately visible when comparing the LSV measurements of fibres with and without cobalt at the given carbonisation temperatures. Especially at carbonisation temperatures of 800 and 900 °C the enhanced *E*_OCP_ is apparent. For fibres carbonised at 900 °C the difference is as large as 70 mV for these particular measurements, while at 700 and 1000 °C the difference is within the margin of error. In general, the fibres with cobalt reach higher current densities, except for very negative potentials vs MMO for the fibres carbonised at 900 °C. The overall current densities seem to increase with carbonisation temperature, with values (at −500 mV vs MMO) starting at 1100 μA·cm^−2^ for fibres carbonised at 700 °C and increasing to 1600 μA·cm^−2^ for fibres carbonised at 1000 °C. Focussing on the fibres carbonised at 1000 °C, the electrode performance of the fibres containing cobalt appears to be more stable compared to the fibres without cobalt, which display a sudden decrease in obtained current densities.

#### Descriptive key parameters derived from LSV measurements

**Figure 9:**
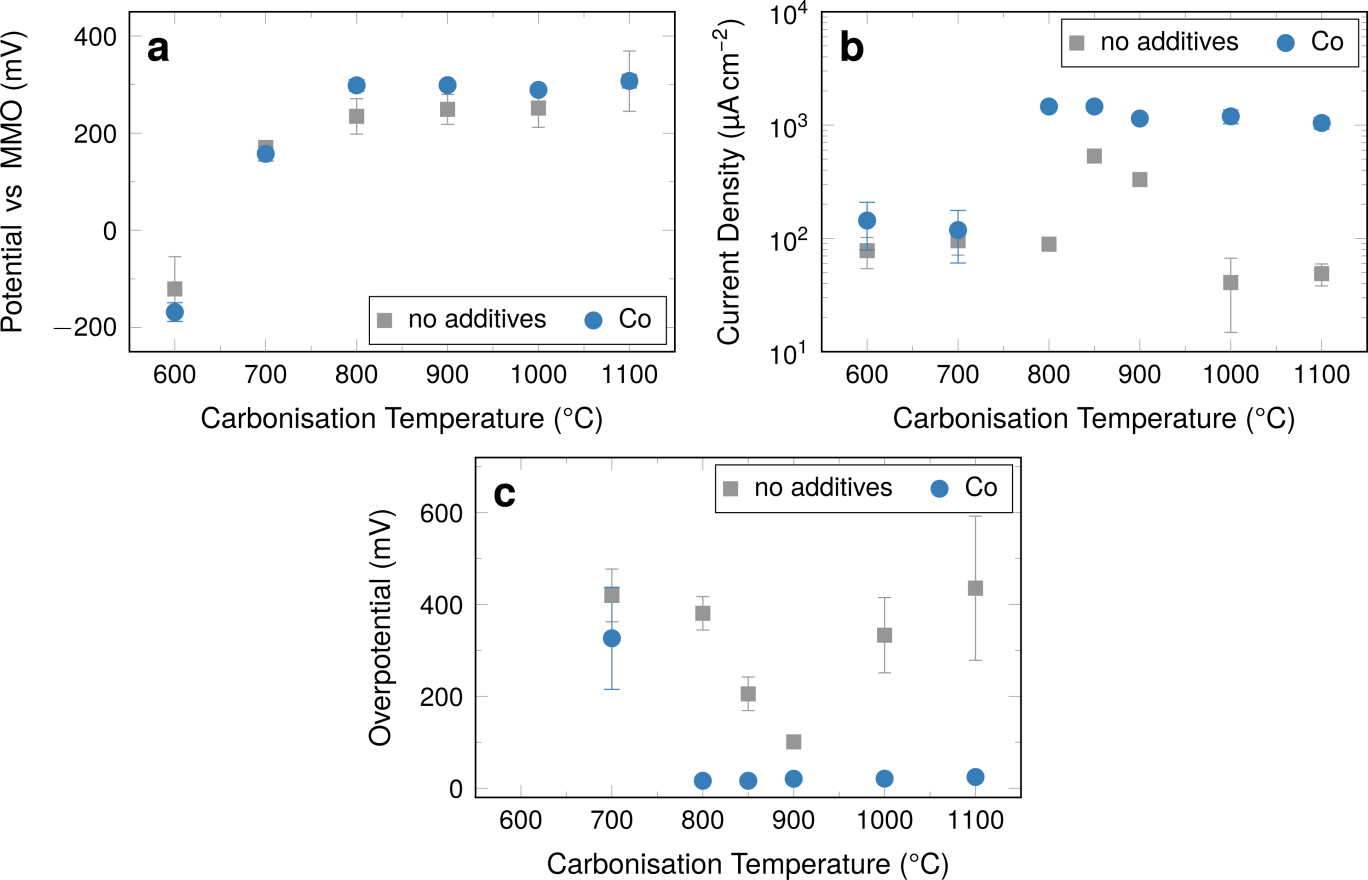
(a) *E*_OCP_ values from LSV measurements. (b) Current density at a given overpotential of 100 mV vs *E*_OCP_. (c) Overpotential at an oxygen reduction current density of 333 μA·cm^−2^. Overpotential values for samples carbonised 600 °C could not be determined, as such high current densities were not obtained from the samples within the investigated potential range. Error bars that are not visible are within the respective symbols.

It is difficult to obtain directly quantitative trends from these measurements. The electrochemical properties of the electrodes can be described quantitatively using the following key parameters: (1) the measured open-circuit potential *E*_OCP_ (Figure 9a), (2) the current density at a given overpotential of 100 mV (Figure 9b), and (3) the required overpotential at a given current density of 333 μA·cm^−2^, which corresponds to a current of 1 mA (Figure 9c).

The open-circuit potential is an indicator for the cell potential of a full cell system. In the ORR case, a higher potential is sought for the air cathode, to increase the overall cell potential. The current density of the system is of relevance to the desired application in terms of available power. The overpotential at a given current density is a measure of the power losses in the system resulting from internal resistances, such as charge transfer resistance and Ohmic resistance relevant to the practical capacity of the system. The resulting values determined in this fashion are summarised in Figure 9.

The *E*_OCP_ values increase with carbonisation temperature between 600 and 800 °C from −190 mV vs MMO to approximately 300 mV vs MMO (Figure 9a). The *E*_OCP_ displays a plateau at this value at higher carbonisation temperatures. The fibres without cobalt overall display a very similar behaviour, although the plateau values of *E*_OCP_ are consistently lower at around 250 mV vs MMO.

The influence of the cobalt catalysts becomes more relevant, when the obtained current densities at an overpotential of 100 mV are considered (Figure 9b). For fibres containing cobalt, there is a distinct increase for carbonisation temperatures between 700 and 800 °C from initially approximately 120 μA·cm^−2^ to approximately 1200 μA·cm^−2^. For fibres carbonised at higher temperatures up to 1100 °C the current density is still around 1000 μA·cm^−2^.

The unmodified fibres display quite constant, low values at approximately 100 μA·cm^−2^, when carbonised at temperatures below 850 °C. A spike in activity occurs for the fibres carbonised at 850 °C. The obtained current density is significantly higher at 530 μA·cm^−2^ than at lower carbonisation temperatures, but lower approximately by a factor of three than in fibres containing cobalt. The current density is significantly lower for fibres carbonised at 900 °C, for which the value is only 330 μA·cm^−2^. In fibres carbonised at 1000 and 1100 °C, values that are even lower than the values found for samples carbonised at 800 °C and below are found.

For fibres containing cobalt, the overpotentials at a current density of 333 μA·cm^−2^ (Figure 9c) display a similar step-like behaviour as the current density at a given overpotential (Figure 9b). The fibres display a comparably high overpotential in samples carbonised at 700 °C at around 330 mV vs *E*_OCP_. The value is significantly lower and very stable for all samples carbonised at 800 °C and higher with overpotentials below 25 mV vs *E*_OCP_.

Samples without additives display a pronounced spike-like behaviour with a minimum of overpotential in fibres carbonised at 900 °C, which is still comparably high at 100 mV vs *E*_OCP_. This pattern is similar to the one found in the current density at a given overpotential. However, the two properties seem to be related only indirectly, as the minimum overpotential is found for fibres carbonised at 900 °C rather than 850 °C.

Overall, fibres containing cobalt carbonised at 800 °C and above display the best performance towards ORR in the half-cell over a larger range of carbonisation temperatures. All values investigated display a plateau-like behaviour, which distinguishes the fibres containing cobalt from the ones without cobalt, which display pronounced extreme values for carbonisation temperatures of around 900 °C for current densities and overpotential. The influence of cobalt on *E*_OCP_ is minimal, but at carbonisation temperatures above 800 °C fibres with cobalt display slightly higher values throughout the investigated range of carbonisation temperatures.

The general electrochemical characteristics of the electrodes can be related to a certain extent to the characteristics of fibre structure and composition described in section “Physical characterisation”, although it is difficult to link individual behaviour found in one analysis directly to the electrochemical behaviour. It is rather necessary to regard the entirety of the insights found in a holistic approach.

The behaviour of the reference fibres without cobalt is governed by two competing properties, nitrogen-content and electrical conductivity. As discussed in detail in a previous study, a higher nitrogen content and a higher conductivity will increase the activity and reduce the required overpotential [12]. However, at increasing temperatures, on the one hand, nitrogen is removed, reducing activity, while on the other hand, the carbon becomes more graphitised increasing electrical conductivity. The optimum of activity is found at temperatures in the range from 850 to 900 °C.

In contrast to the performance progression with a local maximum, fibres with cobalt additives carbonised at temperatures at and above 800 °C display stable performance level values. This is a clear indication of a shift in the relevant catalytic system in favour of cobalt. This means that the nitrogen content, which decreases significantly in this range, and the amounts of catalytically active nitrogen species, which are lower than in the unmodified fibres, seem to have lost their relevance as a determining factor of the system performance. Nevertheless, the formation of significant amounts of turbostratic carbon, leading to a mesopore-like surface structure, seems to add to the performance, as does, of course, the inherent catalytic activity of the employed cobalt species.

#### Electrode stability testing

**Figure 10:**
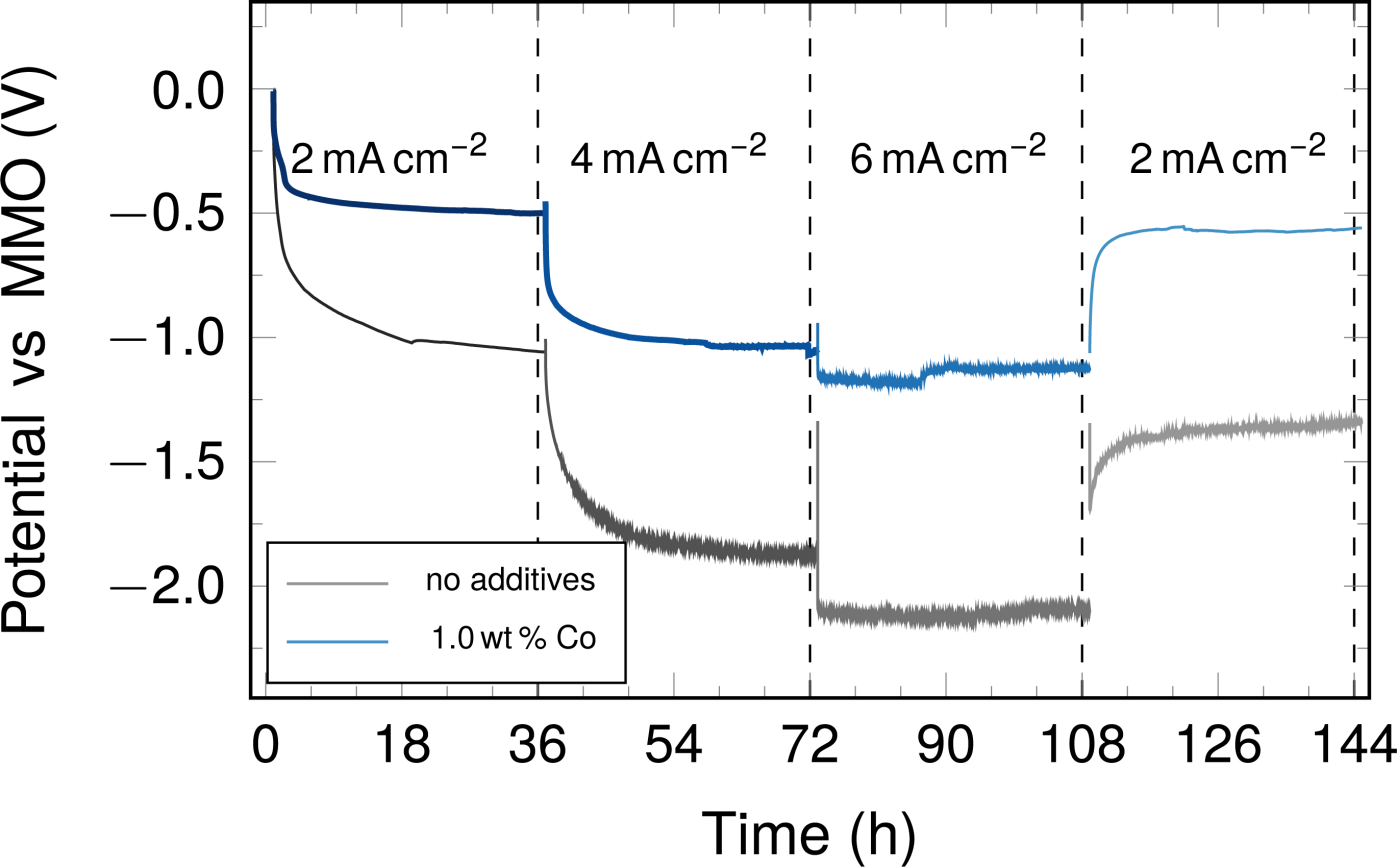
Potential during chronopotentiometry applying constant current densities of 2, 4, 6, and 2 mA·cm^−2^ for 36 h each in the ORR direction.

In applications, performance is important, but electrode integrity and stability for prolonged periods of time under load are typically an issue to be considered. Therefore, the electrodes were discharged with constant currents for an extended period of time, compared to the rather swift potential sweeps described above. Current densities of 2, 4, and 6 mA·cm^−2^ were applied for 36 h to samples carbonised at 900 °C. In a final step the initial current density of 2 mA·cm^−2^ was applied again in order to observe a potential retention (Figure 10).

In the discharge/ORR case the electrodes display very stable potentials, after an equilibration phase of up to 12 h in unmodified electrodes or less in metal-enhanced electrodes, when discharge currents of 2 and 4 mA·cm^−2^ are applied. A stable potential is reached when 6 mA·cm^−2^ are applied after an initial potential drop.

It is apparent that the cobalt enhancement influences the cell potential significantly; at the initial 2 mA·cm^−2^, unmodified fibres exhibit a potential of −1.0 V vs MMO, whereas the cobalt-containing sample exhibits a potential of −0.48 V vs MMO. When higher current densities are applied, the potentials become more negative, although the average potential difference is very low comparing the values at 4 and 6 mA·cm^−2^.

When 2 mA·cm^−2^ are applied again in the last step, the original potential is not reached again for both kinds of electrode, with and without cobalt additive. The final potential is slightly more negative, that is, the overpotential is increased. In the case of the unmodified fibres the potential is 0.3 V more negative, in the case of the cobalt-enhanced fibres, the increase in overpotential leads only to a value that is more negative by 0.07 V. This implies that the effect of the high currents is subdued significantly by the cobalt catalyst. The less negative potential observed in samples containing cobalt compared to samples without any additives is beneficial, as a more positive potential in the half-cell implies a higher overall cell voltage in a full cell.

## Conclusion

In this study, cobalt-particle-decorated PAN-derived carbon fibre networks were prepared using the electrospinning method. The influence of the carbonisation temperature on the graphitisation process and the particle morphology of fibres and cobalt-species was investigated. The fibre mats were applied as free-standing air electrodes in aqueous alkaline metal–air battery half-cells and investigated with respect to electrochemical redox activity and stability.

Microscopic analysis of the resulting fibre mats showed that, with increasing carbonisation temperatures, the mean fibre diameter decreased, whereas the mean cobalt-species particle size increased. The (surface) composition and structure of the particles was determined using both XPS and XRD. The particles were found to be mainly metallic with an oxide layer on their outer surface.

An influence of the cobalt species on the carbonisation process and the resulting microscopic and macroscopic structure was established with Raman, XRD, and XPS. Cobalt was found to enhance the graphitisation and to induce a shift from the formation of mostly crystalline graphite to significant amounts of turbostratic graphite.

The cobalt additive substantially influences nitrogen content and incorporation. The overall nitrogen content is lower, while the relative amount of catalytically inactive pyridinic nitrogen at higher temperatures is higher compared to non-modified fibres.

As for the ORR activity of the fibre mats, it was found that for fibres containing 1.0 wt % of cobalt an increasing carbonisation temperature improves the electrochemical performance only up to 800 °C for the particle-decorated fibre system. The values of the investigated key parameters displayed a plateau in samples carbonised at 800 °C and above. The current density in fibres with cobalt was also found to be about three times as high than the highest value observed in fibres without cobalt additive at 900 °C.

Applying constant currents in the ORR direction for prolonged periods of time showed that the fibre system is very stable within the investigated current density range. It retains most of its activity, also after 6 mA·cm^−2^ have been applied for 36 h. The cobalt-decorated fibre mats display decent performance and stability for ORR, when used directly as free-standing air electrode in a 6 M KOH electrolyte-based half-cell system.

## Supporting Information

Supporting Information features the elemental composition of fibres from elemental analysis and ICP-OES; N 1s, C 1s, and Co 2p XPS spectra for samples with cobalt; and LSV curves for all samples.

File 1 Additional experimental data.
