# Supplementary material for: The effect of cobalt on morphology, structure, and ORR activity of electrospun carbon fibre mats in aqueous alkaline environments
Source: Beilstein J Nanotechnol. 2021 Oct 19;12:1173–86. doi: 10.3762/bjnano.12.87 (PMC8551909; doi:10.3762/bjnano.12.87)
Supplement: File 1 — Additional experimental data. [file Beilstein_J_Nanotechnol-12-1173-s001.pdf]

BEILSTEIN JOURNAL OF NANOTECHNOLOGY

Supporting Information

for

The effect of cobalt on morphology, structure, and ORR activity of electrospun carbon fibre mats in aqueous alkaline environments

Markus Gehring, Tobias Kutsch, Osmane Camara, Alexandre Merlen, Hermann Tempel, Hans Kungl and Rüdiger-A. Eichel

Beilstein J. Nanotechnol. **2021,** *12,* 1173–1186. doi:10.3762/bjnano.12.87

Additional experimental data

License and Terms: This is a supporting information file under the terms of the Creative Commons Attribution License (https://creativecommons.org/licenses/by/4.0). Please note that the reuse, redistribution and reproduction in particular requires that the author(s) and source are credited and that individual graphics may be subject to special legal provisions.
The license is subject to the *Beilstein Journal of Nanotechnology* terms and conditions: (https://www.beilstein-journals.org/bjnano/terms)

1 Elemental analysis results

Table S1: Composition of the fibres without any additives after stabilisation in air at 250 °C for 12 h and subsequent carbonisation in argon at the indicated temperatures and a holding time of 3 h. All values were determined by elemental analysis. Values are given with the standard derivation of three independent measurements of the same sample.

T_C °C	**Carbon** wt %	**Hydrogen** wt %	**Nitrogen** wt %	**Oxygen** wt %	**Sum** wt %
600	65.5±0.33	2.46±(<0.01)	19.8±0.28	1.8±(<0.1)	89.56±0.72
700	70.8±(<0.1)	2.09±0.06	16.1±0.22	10±0.15	98.99±0.5
800	73.8±0.28	1.5±0.08	13.6±0.27	10.9±0.47	99.8±1.1
900	82.1±0.18	1.34±0.03	5.9±0.05	11.4±0.11	100.74±0.37
1000	94.7±0.28	0.54±0.03	3.83±0.12	10.8±0.15	109.87±0.58
1100	78.3±0.3	1.5±0.03	9.29±0.23	4.44±0.2	93.53±0.76

Table S2: Composition of the fibres with initially 1.0 wt % of cobalt after stabilisation in air at 250 °C for 12 h and subsequent carbonisation in argon at the indicated temperatures and a holding time of 3 h. Contents of C, H, N, and O were determined by elemental analysis, Co content was determined by ICP-OES. Values are given with the standard derivation of three independent measurements of the same sample.

T_C °C	**Carbon** wt %	**Hydrogen** wt %	**Nitrogen** wt %	**Oxygen** wt %	**Cobalt** wt %	**Sum** wt %
600	50.8±0.19	2.03±0.01	19.5±(<0.01)	15.1±1.13	12.15±0.07	99.58±1.41
700	58.5±0.43	1.43±(<0.01)	12.9±0.34	13.4±0.3	13.76±0.11	99.99±1.19
800	67.9±0.22	1.3±0.01	8.03±0.08	10.6±0.4	8.98±0.09	96.81±0.8
900	71.4±0.38	1.14±(<0.01)	4.5±0.04	8.78±0.62	12.54±0.16	98.36±1.21
1000	76.4±0.44	0.99±0.02	2.59±0.11	6.83±0.02	12.91±0.19	99.72±0.78
1100	78.8±(<0.1)	0.83±0.07	1.71±0.11	4.89±0.1	12.5±0.2	98.73±0.58

2 XPS spectra overview of samples with cobalt

2.1 Carbon C 1s spectra

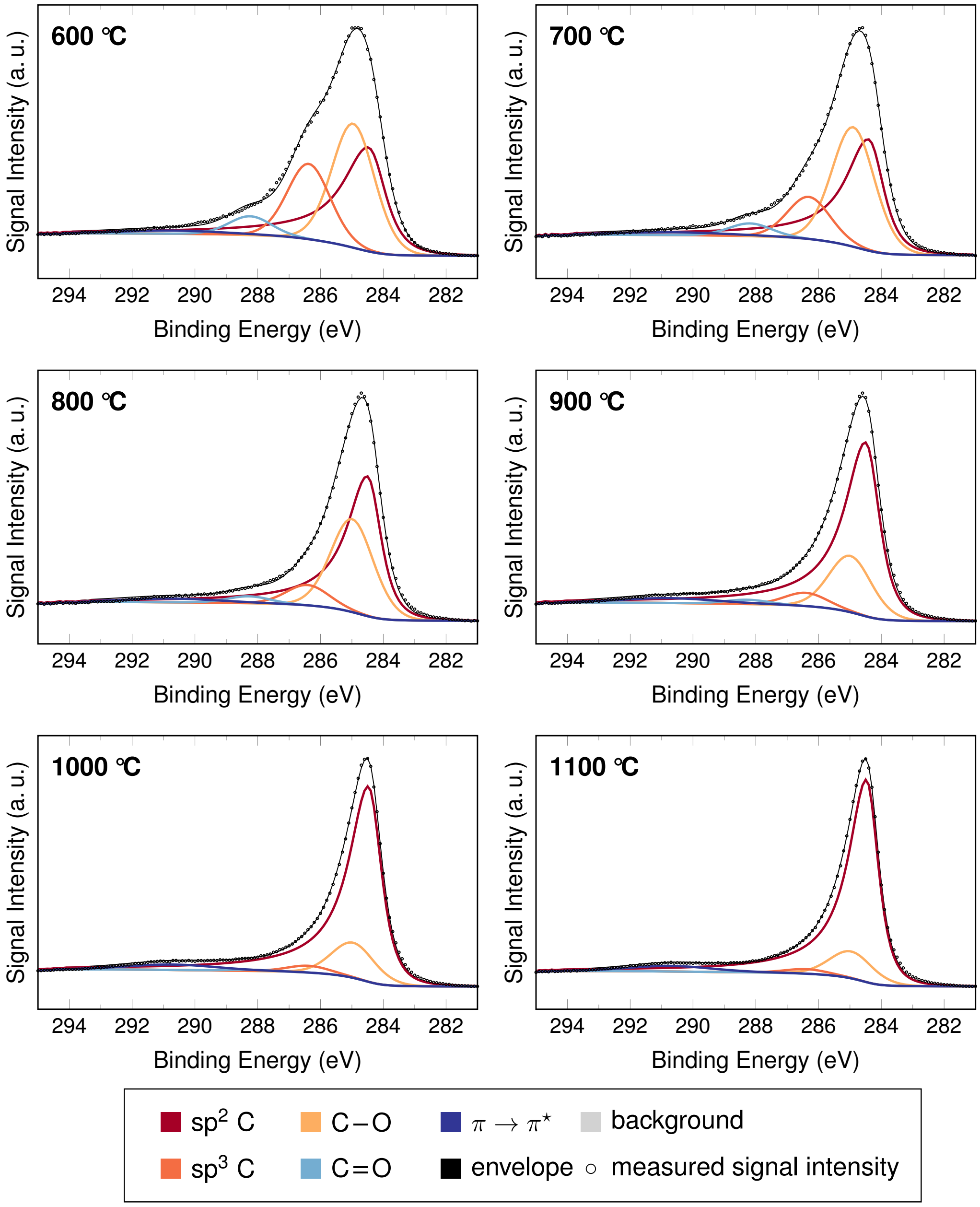

Figure S1: C 1s XPS spectra of samples containing cobalt.

2.2 Nitrogen N 1s spectra

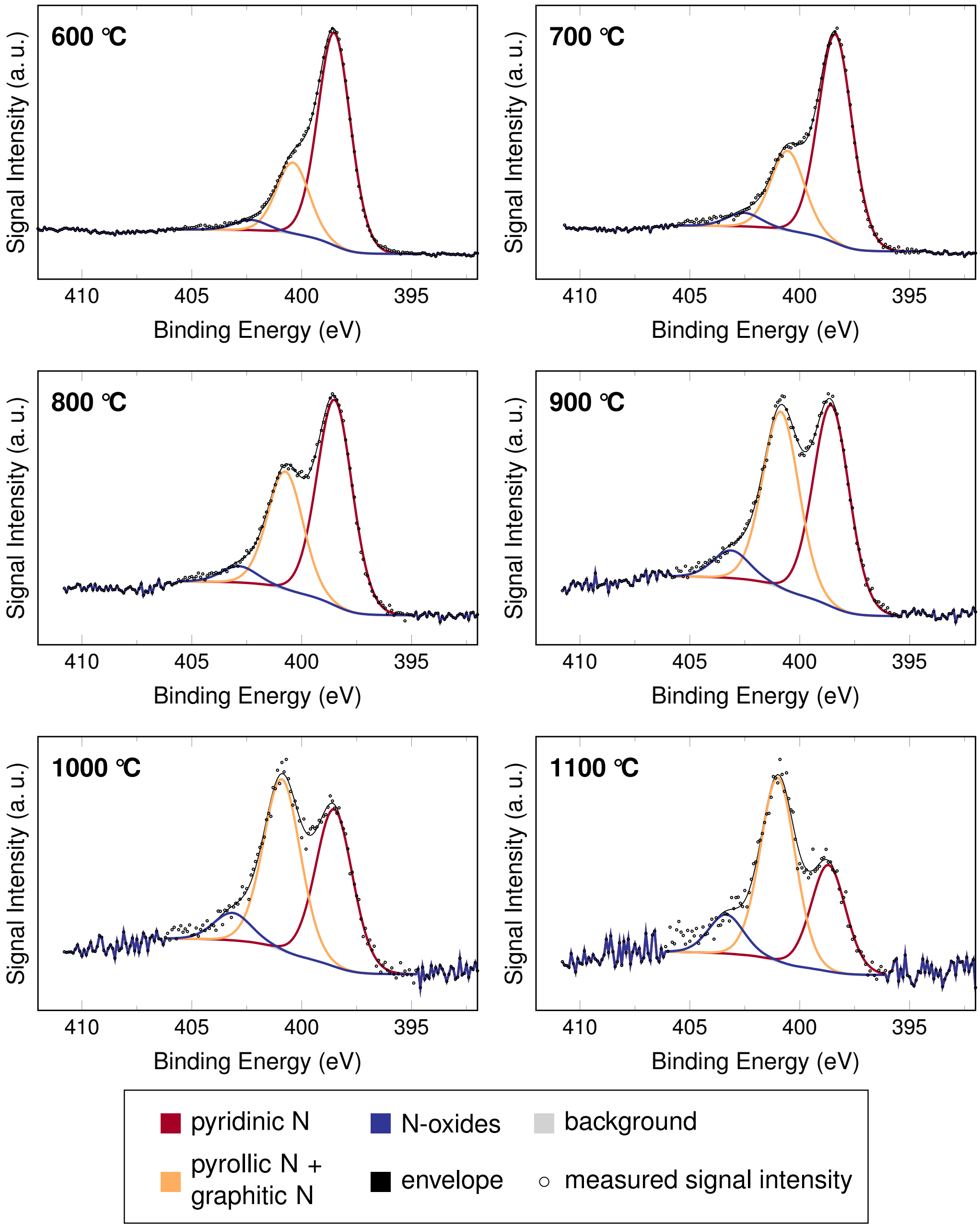

Figure S2: N 1s XPS spectra of samples containing cobalt.

2.3 Cobalt Co 2p spectra

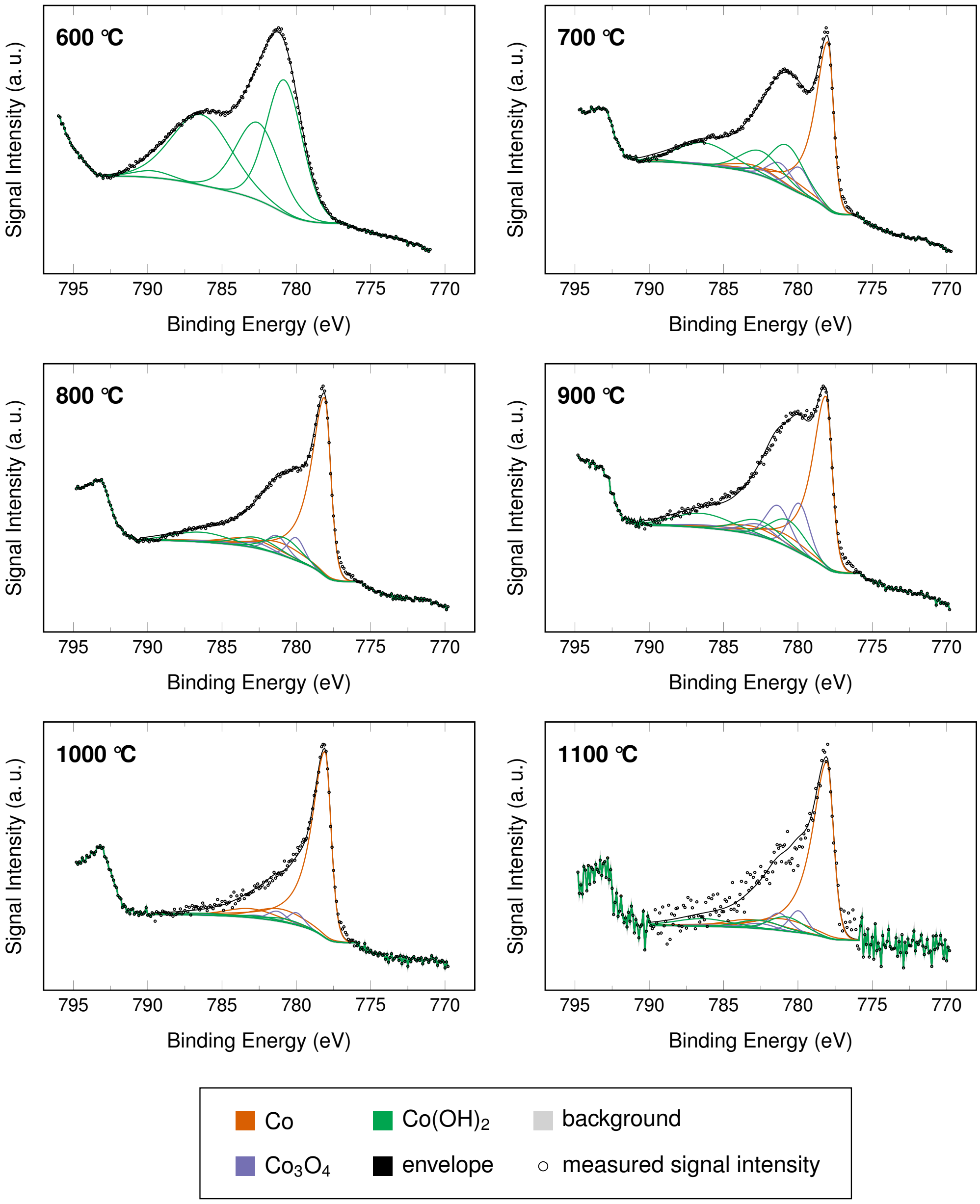

Figure S3: Co 2p XPS spectra of samples containing cobalt.

3 LSV measurement data

3.1 Samples with cobalt

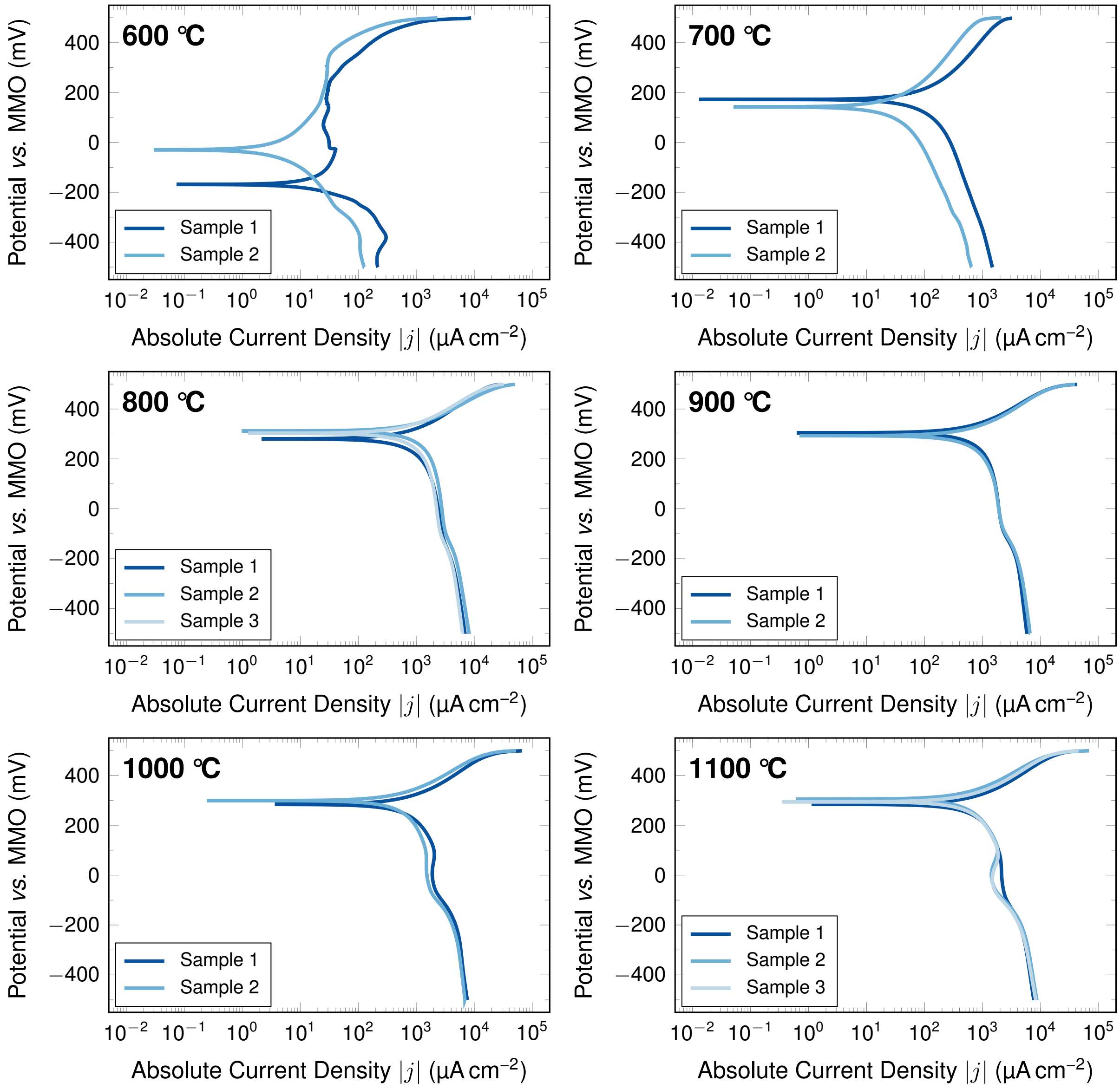

Figure S4: Linear sweep voltammetry ORR measurements for independent samples spun with 1.0 wt % cobalt, carbonised at the indicated temperatures.

3.2 Samples without cobalt

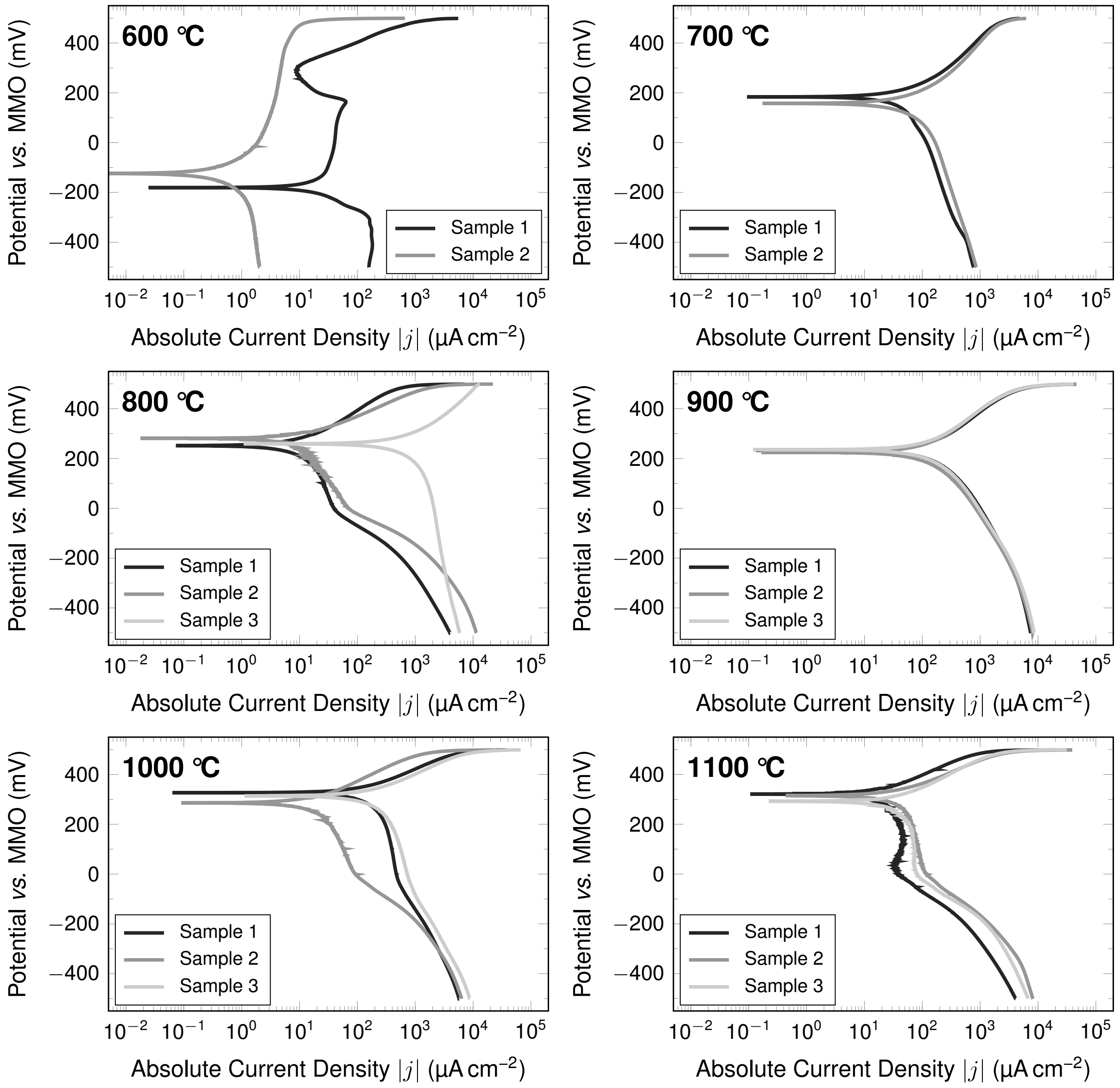

Figure S5: Linear sweep voltammetry ORR measurements for samples spun without additives, carbonised at the indicated temperatures.
